# Supplementary material for: On-chip bacterial foraging training in silicon photonic circuits for projection-enabled nonlinear classification
Source: Nat Commun. 2022 Jun 30;13:3261. doi: 10.1038/s41467-022-30906-3 (PMC9247170; doi:10.1038/s41467-022-30906-3)
Supplement: Supplementary file 1 — Supplementary Information [file 41467_2022_30906_MOESM1_ESM.pdf]

Supplementary Information

On-chip bacterial foraging training in silicon photonic circuits for projection-enabled nonlinear classification

Guangwei Cong*, Noritsugu Yamamoto, Takashi Inoue, Yuriko Maegami, Morifumi Ohno, Shota Kita, Shu Namiki, and Koji Yamada

*To whom correspondence should be addressed; e-mail: gw-cong@aist.go.jp

1. Projection-enabled classification vs ANN

1.1 ANN examples vs projection-based program for XOR classification

The projection-enabled nonlinear classification is distinguished from the traditional ANN (artificial neural network, here an ANN means a multilayer perceptron which can be expressed as $\mathbf{y} = \mathbf{w}f(\ldots\mathbf{w}f(\mathbf{wx} + \mathbf{b}) + \mathbf{b})$, where f is a nonlinear activation function and $\mathbf{w}$ and $\mathbf{b}$ are weight parameters, $\mathbf{x}$ is the input vector and x is an element of the vector $\mathbf{x}$). For understanding the difference, we prepare three ANN examples and a program to emulate what the PPC does for XOR classification. This program directly uses the projection generated by the phase-amplitude relation of MZI (Mach-Zehnder interferometers). These examples are shown in Supplementary Fig. 1. All ANNs use a 2×4×2 structure (2 input nodes, 4 hidden nodes, 2 output nodes). Supplementary Fig. 1(a) shows the ANN without nonlinear activation functions. XOR cannot be separated by this purely linear ANN. This explains why XOR is usually regarded as a linearly inseparable problem. Supplementary Fig. 1(b) shows the ANN using ReLU (rectified linear unit) activation functions, which also cannot classify XOR. Supplementary Fig. 1(c) shows the ANN using Sigmoid activation functions, which can successfully classify XOR. The reason why the ANN using Sigmoid can separate XOR, but that using ReLU cannot, is mainly due to the non-zero output (0.5) for a zero input of Sigmoid function (0→0.5 infinite amplification), which is important for recognizing the 00 pattern with a same value as 11. However, ReLU remains linear in essence for either $(-\infty, 0]$ or $[0, \infty)$ even though it is a nonlinear function for the whole region. Supplementary Figs. 1(d) and 1(e) show the projection-based programs for two normalized phase values of π and $\pi/2$ for the bit 1, respectively. Each bit pattern is mapped to a 4-dimension complex vector by using the phase-amplitude nonlinearity of a 2×2 MZI in the equation (Eq. S1) and an input optical field of [1, 0]. This is a nonlinear map because the map function G from the input vector space ($x \in R$) to the projected space ($x' \in C$) does not satisfy the Additivity condition, $G([0,0] + [1,1]) \neq G([0,0]) + G([1,1])$. Afterwards, a linear transformation in complex domain completes separation and the squared modulus (corresponding to optical power) gives out the label. Obviously, the projection is not unique as shown in Supplementary Figs. 1(d) and 1(e), which is determined by the normalized phase range and data input scheme. Direct mapping operation on the data using a pre-chosen nonlinear function and a followed linear separation is a more SVM (supporting vector machine) – like idea than ANN (Refs. [1–4]). This idea can be understood schematically in Supplementary Fig. 2 which depicts a quadratic mapping and a sinusoidal mapping which can be implemented by using the phase-amplitude relation of MZI. Since the mapping function can be

constructed in many ways as seen in Fig. 1b–1d in the main text by inputting the data into cascaded MZIs (sinusoidal multiplications), such a direct treatment on the data to generate projection-assisted classification effect cannot be fully covered by the traditional ANN frame. In addition, Supplementary Fig. 1(f) shows the comparison on the accuracy convergence between the ANN in Supplementary Fig. 1(c) and the projection-based programs in Supplementary Figs. 1(d) and 1(e), which shows that Supplementary Figs. 1(d) and 1(e) have a fast convergence than Supplementary Fig. 1(c).

$$\mathbf{T}(x) = \frac{1}{2}\begin{bmatrix} e^{ix}-1 & i(e^{ix}+1) \\ i(e^{ix}+1) & 1-e^{ix} \end{bmatrix} = ie^{ix/2}\begin{bmatrix} sin(x/2) & cos(x/2) \\ cos(x/2) & -sin(x/2) \end{bmatrix} \qquad \text{(Eq. S1)}$$

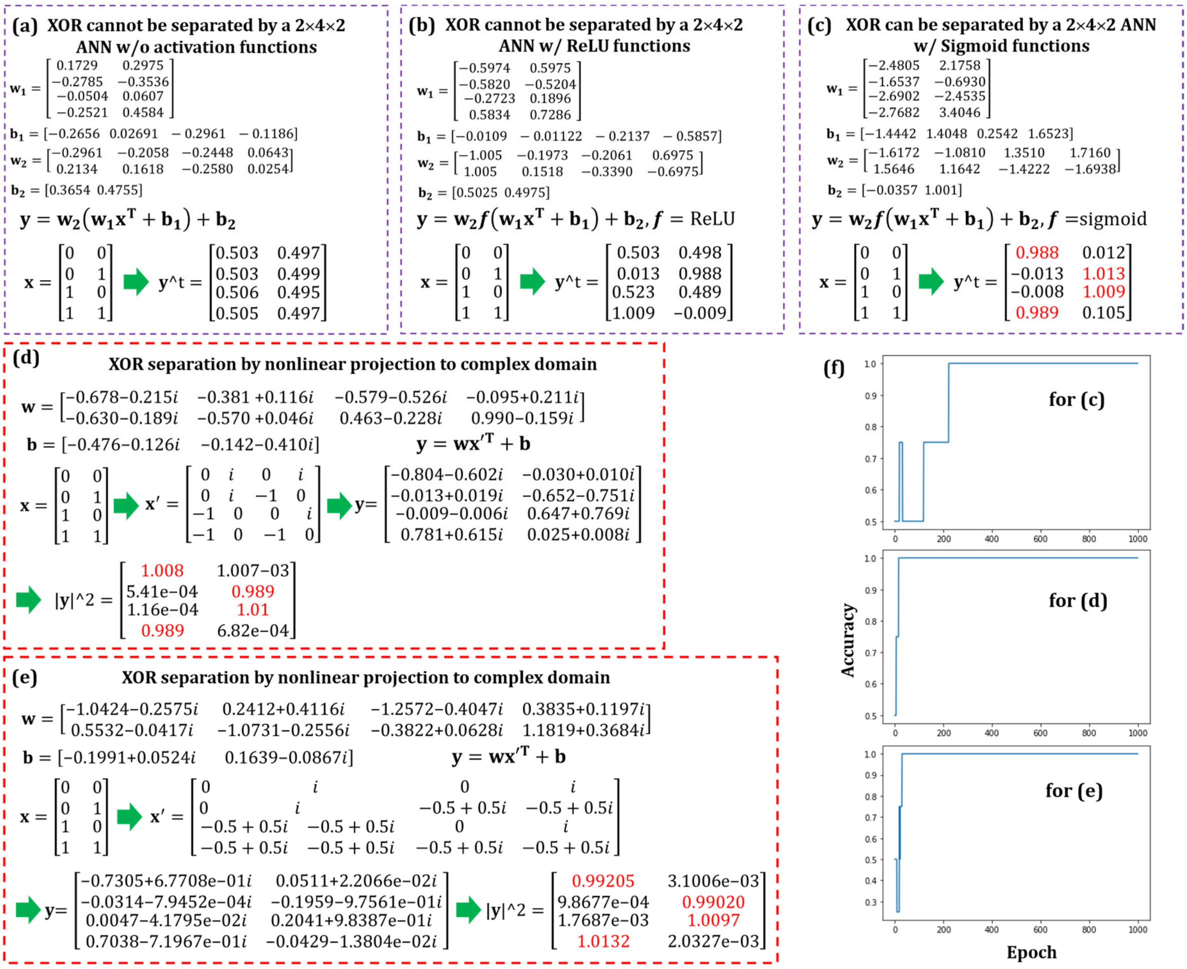

Supplementary Fig. 1. Three examples of simple 2×4×2 ANNs for XOR classification: (a) purely linear without activation functions; (b) with ReLU; (c) with Sigmoid. Projection-enabled XOR separation using the phase-amplitude nonlinearity in MZI to emulate the PPC device: (d) π and (e) $\pi/2$ for the phase of bit 1. (f) Training curves of accuracy of the ANN in (c) and the projection-based programs in (d) and (e).

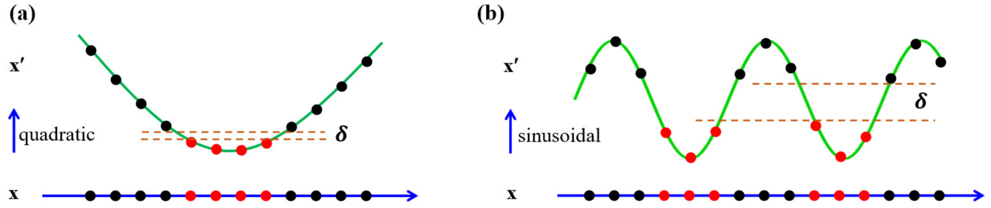

Supplementary Fig. 2. Schematic of mapping functions: (a) quadratic function usually used in SVM; (b) sinusoidal function which can be implemented by photonic devices in the complex amplitude domain.

1.2 SVM-like principle's instantiations

SVM is a supervised learning algorithm, which seeks a hyperplane with a maximum margin in a higher dimensional projected space to separate data. Taking the case in Supplementary Fig. 1(d) as an example, even though detecting the optical power involves in a function of squared modulus, the classification is ready done in multiple dimensional complex vector space as seen from the distance to the origin of elements of **y**. As explained in the main text (section Model explanation), from the established equation $\mathbf{wx'}^T + \mathbf{b} = \mathbf{y}$ to $\mathbf{wx'}^T + \mathbf{b} = 0$ by changing $\mathbf{x'} \leftarrow [\mathbf{x'}, -\mathbf{y}]$ and $\mathbf{w} \leftarrow [\mathbf{w}, [1,1]^T]$, we can construct a hyperplane in a five dimensional complex space for each column of $\mathbf{y_i}$ (corresponding to a plane that does a one-vs-other classifier). Then, the samples can be expressed as $\mathbf{x_d} = [\mathbf{x'}, [0,1,1,0]]$ for the column 1 and the distance $\mathbf{d}(\mathbf{x}, P)$ from $\mathbf{x_d}$ to the plane P: $\mathbf{wx'}^T + \mathbf{b} = 0$ can be calculated as:

$$\mathbf{d}(\mathbf{x_d}, P) = \frac{|\mathbf{wx_d} + \mathbf{b}|}{||\mathbf{w}||} = \frac{1}{||\mathbf{w}||}$$

$$|\mathbf{wx_d} + \mathbf{b}| = \left|(-0.678-0.215i, -0.38+0.116i, -0.579-0.526i, -0.095+0.211i, 1)\begin{pmatrix} 0 & i & 0 & i & 0 \\ 0 & i & -1 & 0 & 1 \\ -1 & 0 & 0 & i & 1 \\ -1 & 0 & 0 & -1 & 0 \end{pmatrix}^T + (-0.476-0.126i)\right| =$$

(1 1 1 1), for column 1 of **y**

$$|\mathbf{wx_d} + \mathbf{b}| = \left|(-0.630-0.189i, -0.57+0.046i, 0.463-0.228i, 0.990-0.159i, 1)\begin{pmatrix} 0 & i & 0 & i & 1 \\ 0 & i & -1 & 0 & 0 \\ -1 & 0 & 0 & i & 0 \\ -1 & 0 & 0 & -1 & 1 \end{pmatrix}^T + (-0.142-0.410i)\right| =$$

(1 1 1 1), for column 2 of **y**

Obviously, all data points have the same distance of 1/||**w**|| to the plane, which indicates that the margin is maximized and all XOR pattens are the support vectors. Similar condition is also satisfied for the case of Supplementary Fig. 1(e). Thus, training using the power as target is essentially same as using the distance in complex space. Training the power of **y** is equivalent to maximizing the distance, i.e., minimizing the ||**w**||^2, which is the same optimization target as used in the SVM algorithms (as shown by Eq. 1.24 in Ref. [3]).

1.3 SVM-like kernel description

SVM usually adopts kernel technique to save the computational cost for nonlinear mapping (Refs. [3,4]); and then the whole algorithm can be kept same as linear classifiers except for replacing the dot product by kernel functions. The PPC does not directly use the kernel method in training, while as explained in the section of Model explanation in the main text, training this photonic chip can produce an implicit equation related to the kernel matrix. Here, taking XOR as an example, we show that the PPC is equivalent to the kernel method based on dot product calculation in the projected space. Once trained, the device can be expressed as $\mathbf{y} = \alpha K(\mathbf{x},\mathbf{v}) + \beta$, where the kernel function K is related to the mapping function G as shown in Eq. S2 (*denotes the conjugate transpose), by considering both Eq. (28) and Eq. (33) in the main text for simpleness. The equations of G are shown in Eqs. (1)–(24) in the main text. For Supplementary Fig. 1(d), we can derive out the **K** matrix as shown in Eq. S3 from the dot product of the

projected vector **x'** for each pair of two samples.

$$K(\mathbf{x}, \mathbf{v}) = |\langle G, G^* \rangle|^2 \qquad \text{(Eq. S2)}$$

$$\mathbf{K} = \begin{pmatrix} 4110 \\ 1401 \\ 1041 \\ 0114 \end{pmatrix}, \quad \boldsymbol{\alpha} = \begin{pmatrix} -0.5 \\ 0.5 \\ 0.5 \\ -0.5 \end{pmatrix} \quad \beta = [0,0,0,0] \qquad \text{(Eq. S3)}$$

It is easy to find α as shown in Eq. S3 to make **y** = [−1,1,1, −1], indicating classification according to the sign or values (e.g., using β = [1,1,1,1]). Similar classification via constructing **K** matrix can also be done for Supplementary Fig. 1(e). The K function interprets a similarity between two projected vectors. Thus, classification in the PPC is equivalent to constructing an implicit kernel function by which classification becomes an optimization problem with a similar procedure as SVM (see Model explanation in the main text).

2. Experimental details

2.1 Control parameter

For two 40-channel direct-current (dc) sources used in our experiment, we control the DAC (digital-to-analog) value for each channel to control the output voltage. They are of 16-bit resolution. It is the DAC value at each channel that the algorithm optimizes as the experimental weight parameter. The relation between voltage and current and the DAC was measured for all heaters to evaluate the phase shifter efficiency. The DAC-voltage relation is shown in Supplementary Fig. 3(a) for two heaters: 1st one at the layer 1 (heater for the first bit of XOR) and 4th one at the layer 2 (the fourth parameter for Iris) (the layer number is shown in Fig. 1a in the main text). Due to the apparatus property of dc sources, there are no effective voltage output for the DAC less than ~2000. Around 2000, there are small nonlinear voltage outputs. After that, the voltage is linearly increased with the DAC with a resolution of 0.00044 V/bit.

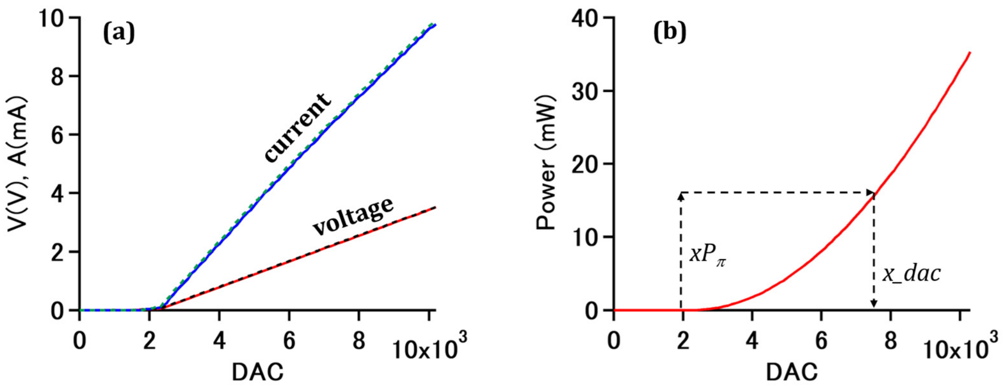

Supplementary Fig. 3. (a) Measured voltage and current vs the DAC for two heaters. Solid: 1st heater at the layer 1. Dotted: 4th heater at the layer 2 (the layer number is the column number of MZI in Fig. 1a in the main text). (b) Measured power in relation of the DAC for the 1st heater at the layer 1. The DAC of input data *x* was prepared by interpolation.

The current is almost linearly dependent on DAC, while a weak heating-induced resistance increase can be noticed. But this does not influence the on-chip training because the algorithm essentially optimizes the power induced phase shift and dynamically takes any possible variations into the training process,

without caring about how uniform or variable the resistance is. Note that the small current difference in Supplementary Fig. 3(a) results from a small difference in resistance of the heaters.

The algorithm directly controls the DAC in the experiment, and since the DAC and voltage are linear to each other, we use the voltage instead of DAC in the main text and in the following discussions for conciseness.

2.2 Input data for experiment

Here we explain how the input data is prepared. As said in the manuscript, all input data x (XOR, Iris, etc.) are normalized to the phase in unit of π radians. We need the corresponding DAC of the normalized input data x. Since the phase is linear to the power, we can convert x to DAC (=voltage) by performing interpolation from xP_π to x_dac using the power-DAC (voltage) curve, as shown in Supplementary Fig. 3(b), where P_π is the π-shift power (measured values are shown in Section 2.3 below). For each of x, this interpolation is done using the power-DAC curve of its corresponding heater. The DAC biases (off-state reference point for interpolation) are included as the training parameters.

2.3 Phase shifter efficiency

The measured resistances and π-shift powers (P_π) are shown in Supplementary Figs. 4(a) and 4(b), respectively, for all heaters. The average resistance is about 347 Ω. The average P_π is about 17.2 mW and the standard deviation is 0.66 mW, showing high uniformity. Within the DAC tuning range, the maximum powers of all heaters are about 35–38 mW (slightly varies with the heaters due to resistance difference), covering a phase tuning range of [0, ~2.1π].

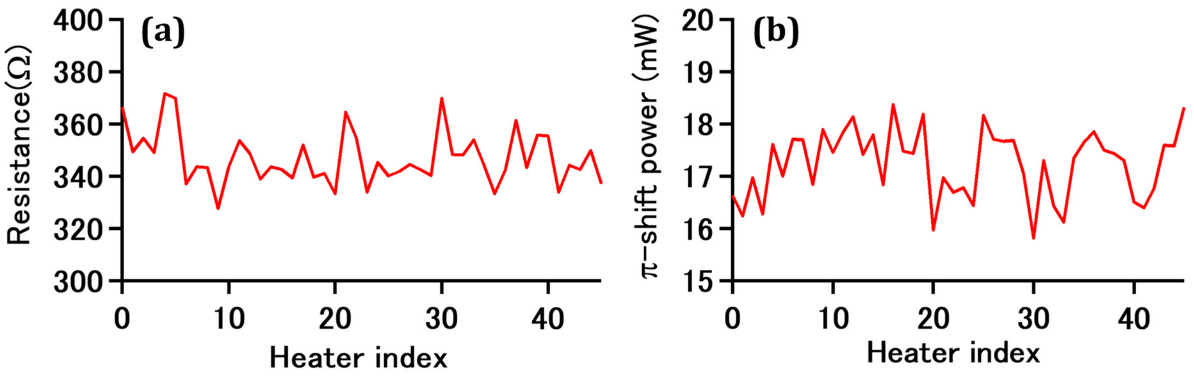

Supplementary Fig. 4. Measured resistance (a) and π-shift powers (b) of all heaters. Heater index is counted from the left column of MZI and from the top for each column.

3. Additional results for XOR, other single and combinational Boolean logics

3.1 Experiment and simulation comparison of BFO training for XOR

In experiment, the algorithm controls the voltage, while in simulation, the algorithm controls the phase. Supplementary Fig. 5(a) shows another BFO training experiment under different conditions, showing even better convergence than that used in Fig. 3 in the main text, which proves that the BFO training is not so strict on the condition and has higher success rate than RMSprop even with a larger voltage step. The MSE of BFO training is compared between the experiment (from Fig. 3 in the main text) and two

simulation cases using 10 (sim 1) and 5 (sim 2) chemotaxis loops in Supplementary Fig. 5(b). After 50 epochs, the experimental MSE is almost same as that of sim 2, both slightly larger than that of sim 1. The simulated optical power maps are shown in Supplementary Fig. 5(c). We have confirmed that setting the parameters is not strict in both simulation and experiment for BFO. No matter constant (e.g., $\Delta\phi = 0.03\pi$) or adaptive steps (e.g., $\Delta\phi$ = $0.03\pi\delta$ or $0.05\pi\delta$) were used, correct classification could be reached with only a difference in optical power like the figures in Supplementary Fig. 5(c), corresponding to a small difference in the final MSE. Different from simulation, the experiment suffers noise influence, thus, we need a slightly larger step at beginning and smaller ones with progressing for better convergence, for which we use an adaptive voltage step in experiment. The simulation uses the exact same structure in Fig. 1a in the main text. Algorithm comparison between BFO and RMSprop in simulation is given in Supplementary Section 7.

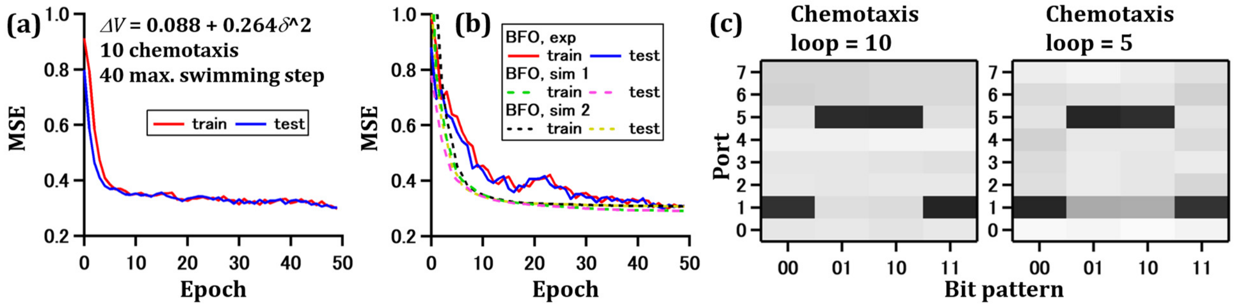

Supplementary Fig. 5. (a) Another BFO training experiment of XOR classification with different conditions. (b) Comparison on MSE of BFO training between experiment and simulation for XOR classification. exp: experiment. sim: simulation. (c) Simulated optical power map of XOR classification using different chemotaxis loops.

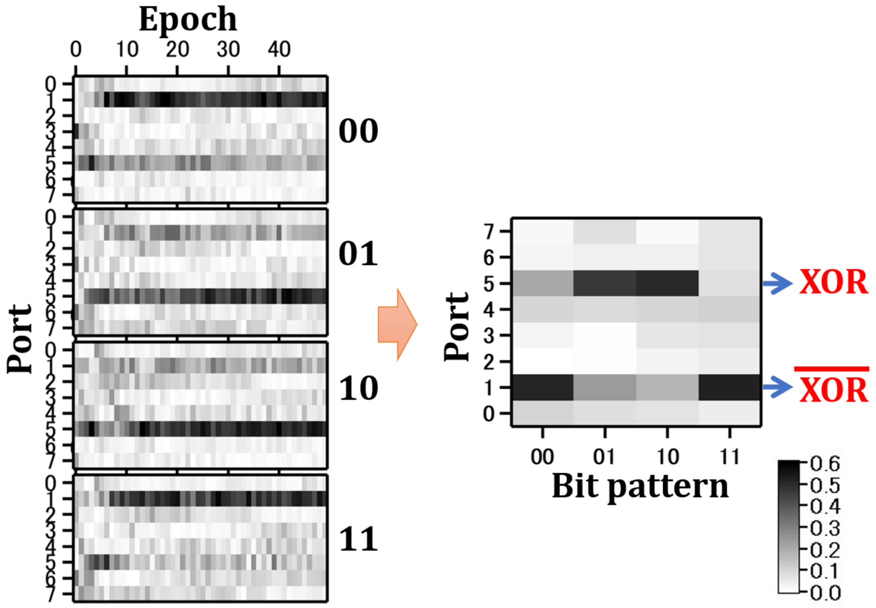

Supplementary Fig. 6. Measured optical power evolution with epoch in BFO training for each bit pattern of XOR.

3.2 Convergence process in XOR experiment

Supplementary Fig. 6 shows the optical power evolution at each output port in BFO training experiment of XOR. After ~10 epoch, the correct power configuration at the target ports was achieved for each bit pattern. For comparison, the simulated optical convergence process is also shown in Supplementary Fig.

7. Optical power evolution in Supplementary Fig. 7(a) reasonably interprets the experimental results in Supplementary Fig. 6. How the light propagates inside the device after training is shown in Supplementary Fig. 7(b). When we switch the input bits (see the column of MZI layer index = 2 since the input light is displayed at the index 0 in this figure), the light propagates to the corresponding ports according to the XOR logic (port 5 = XOR(b_1,b_2), port 1 = the bar of XOR). (b_i is the bit of 0 or 1).

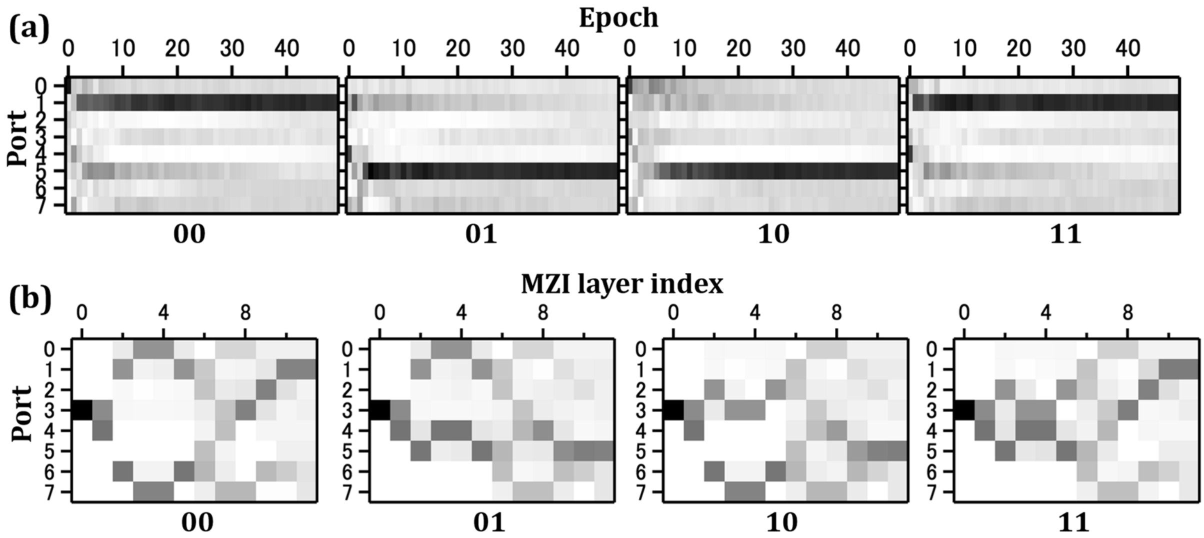

Supplementary Fig. 7. (a) Simulated optical power evolution with epoch in BFO training for each bit pattern of XOR. (Color label max = 0.6 set same as Fig. 6). (b) Optical propagation inside the device after 50 training epochs. (color label max = 1, the input optical power =1, the optical power was taken at the output ports of each MZI).

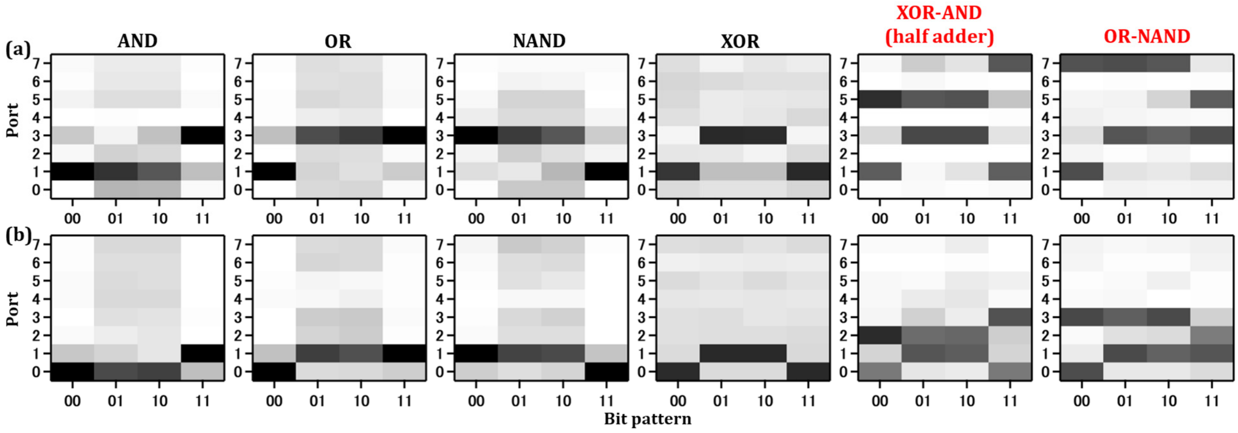

Supplementary Fig. 8. 2-bit logic operation (AND, OR, NAND, XOR) and combinational logic operation (XOR-AND, OR-NAND) trained by BFO (color label max = 0.6): (a) using port 1 (5) and 3 (7) as X = 0 and 1, respectively. (b) using port 0 (2) and 1 (3) as X = 0 and 1, respectively. X = B(b_1, b_2). B represents a Boolean logic operation.

3.3 Single and combinational 2-bit logic operation (simulation)

Beside XOR, various Boolean logic operations can be performed in our device. As shown in Supplementary Fig. 8, by reconfiguring the phase weights by BFO, we can achieve AND, OR, and NAND. In real application, the differential operation between two target ports can be adopted. More importantly, combinational logics can be implemented simultaneously in this single device, such as XOR-AND, and OR-NAND, by assigning two ports for each 0 and 1 logic values. The port assignment is also not unique as seen from Supplementary Figs. 8(a) and 8(b). The XOR-AND logic works as a half adder, for which the

port 3 in Supplementary Fig. 8(a) (or 1 in Supplementary Fig. 8(b)) outputting the sum and the port 7 (or 3) outputting the carrier. The realization of multiple logics in one photonic device could be used for performing advanced optical computing.

3.4 Single and combinational 2-bit logic operation with drop out

As mentioned in the main text, in the device, we use an MZI-mesh-based (Clements' topology in Ref. [5]) interferometer circuit to do matrix transformation for the projected vector. For a $N \times M$ (if $M<N$) matrix transformation, when we use all ports in training, we impose an additional condition of energy conservation to this matrix transformation. This condition is automatically satisfied in unitary transformation, but this constraint is not guaranteed in an arbitrary matrix transformation. Thus, the configured matrix using all ports is using a $N \times N$ unitary transformation to approximate a $N \times M$ arbitrary matrix transformation. This approximation is sufficient for simple classification tasks, but it will slightly decrease the accuracy for complex nonlinear classification. Therefore, discarding some ports (drop out) is necessary to further enhance classification performance. Drop out was also used in photonic neural network as seen in Refs. [6, 7]. In the convolutional neural network programs, random drop out is usually adopted to suppress over-fitting and to improve model generalization capability. We examine the effect of drop out for bit pattern recognition. As shown in Supplementary Fig. 9, the bit contrast can be improved compared to using all ports, but this is at the cost of scarifying some optical power.

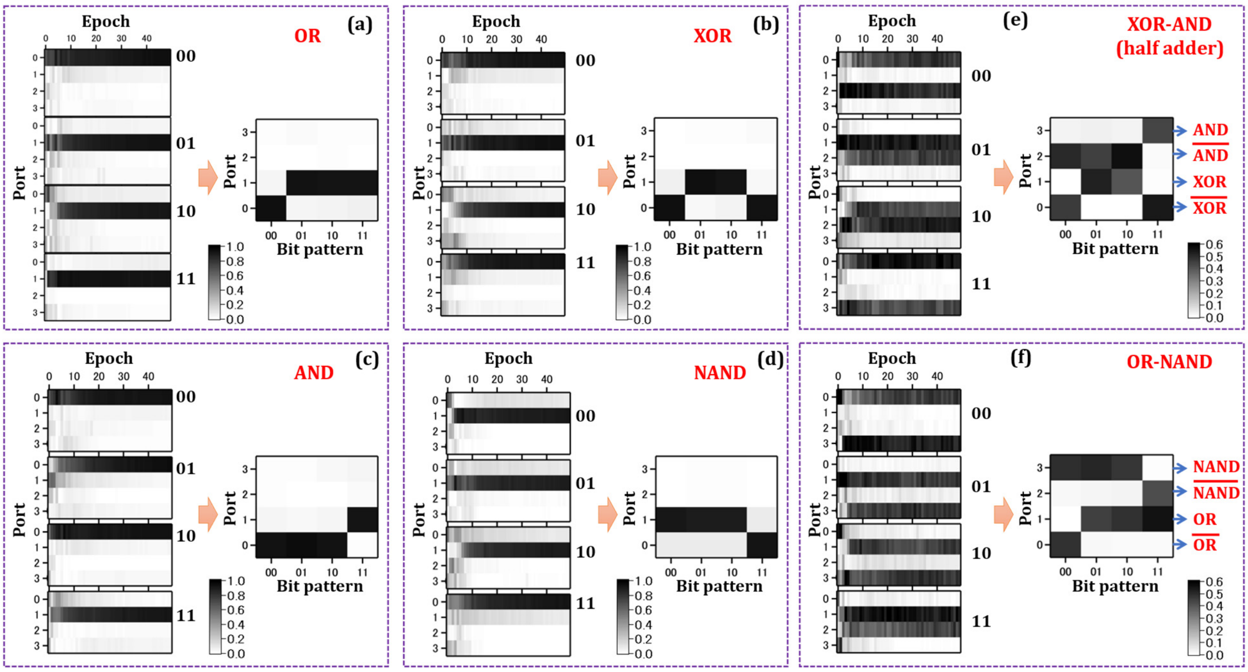

Supplementary Fig. 9. BFO training of XOR with drop out. The ports (0→3) are used and the ports 0 (2) and 1 (3) stand for X = 0 and 1, respectively. X = B(b_1, b_2). B represents a Boolean logic operation. The powers are normalized ($p_i/\Sigma p_i$) for dropped-out ports. Power evolution vs epoch and the final power map for: (a) OR; (b) XOR; (c) AND; (d) NAND; (e) XOR-AND; (f) OR-NAND.

4. Additional results for Iris classification

4.1 Experiment and simulation comparison for Iris classification

Here we compare the experimental and simulated results for each algorithm, BFO and RMSprop.

Supplementary Figs. 10(a) and 10(b) compare the measured and simulated accuracy and MSE of BFO, respectively, so do Supplementary Figs. 10(c) and 10(d) compare those of RMSprop. For each algorithm, the experimental training curves of accuracy and MSE are in good consistence with the simulated ones. Thus, the current experimental setups of parameters (see Methods in the main text) are comparable to those used in simulation. In fact, in simulation, we could adopt more fine steps of phase for BFO and for gradient evaluation of RMSprop, however, it is difficult to adopt so fine steps of voltage in experiment due to the possible noise influence.

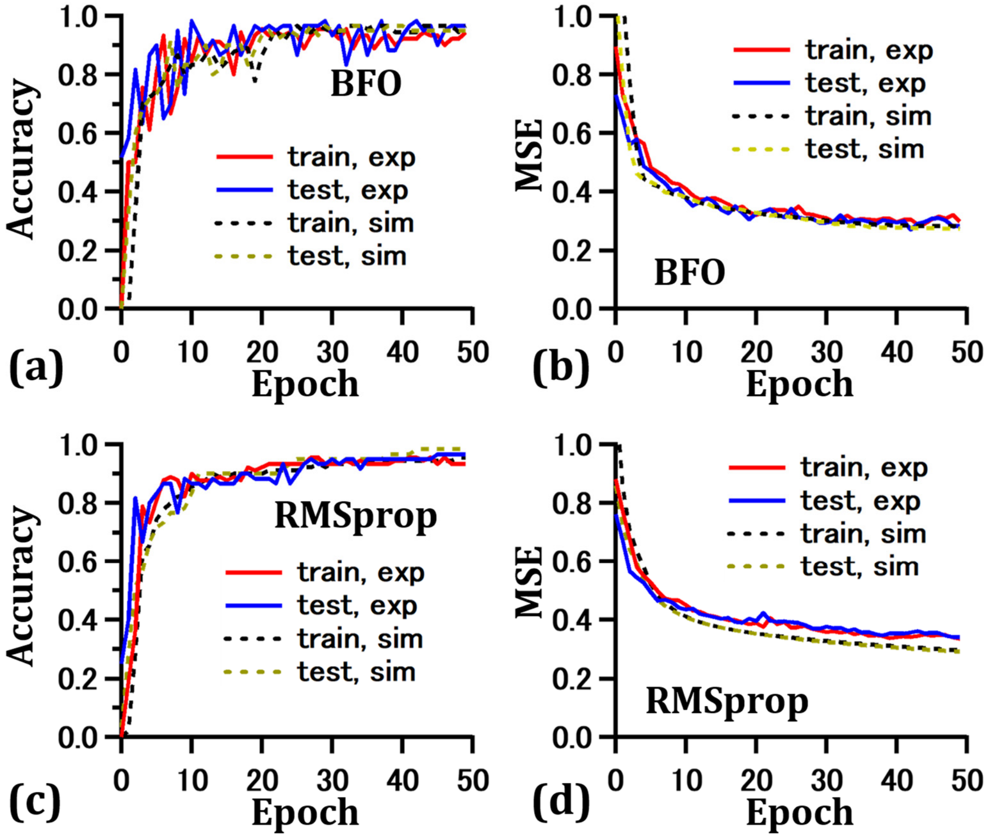

Supplementary Fig. 10: Comparison between experiment and simulation for each algorithm for Iris classification experiment: (a) Accuracy and (b) MSE of BFO; (c) Accuracy and (d) MSE of RMSprop.

4.2 Solution of voltage weights and power consumption: BFO vs RMSprop

Here we show the final voltage weights (i.e., solution of phase distribution) obtained by BFO and RMSprop training, from which we can calculate their power consumption. For Iris classification (corresponding to Figs. 5(a) and 5(b) in the main text), Supplementary Fig. 11 shows the voltages of all heaters and their corresponding power for both BFO and RMSprop. Despite of voltage differences at some heaters, BFO and RMSprop show similarity in the whole profile, confirming the consistency with each other. We sum up the powers at all heaters for each algorithm and obtain the total powers of about 364.5 and 358.7 mW for BFO and RMSprop, respectively. This measurement using wire-bonded chip is more correct than our previous estimation (480 mW) using two 40-pin probes that could impose additional contact resistances.

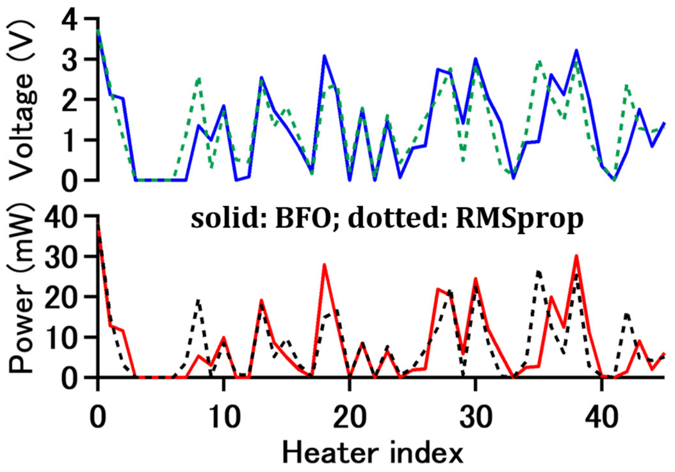

Supplementary Fig. 11: Voltage weights and corresponding electrical powers obtained by BFO and RMSprop training for Iris classification.

4.3 An example of ANN for Iris classification

An ANN model was programmed based on PyTorch (https://pytorch.org/), which consists of two linear layers and a ReLU activation layer. The output is the Softmax function. The training curve of accuracy is shown in Supplementary Fig. 12, showing a 96.67% (max) verification accuracy for the same test set used in our above on-chip training experiment.

A PyTorch-based 4×5×3 ANN

```
class AnnNet(nn.Module):
    def __init__(self, innum, nodenum, outnum):
        super(AnnNet, self).__init__()
        self.linear1 = nn.Linear(innum, nodenum)
        self.linear2 = nn.Linear(nodenum, outnum)
    def forward(self, x):
        x = self.linear1(x)
        x = F.relu(x)        #cancel this for pure linear
        x = self.linear2(x)
        y = F.softmax(x, dim = 1) # cancel this for pure linear
        return y
```

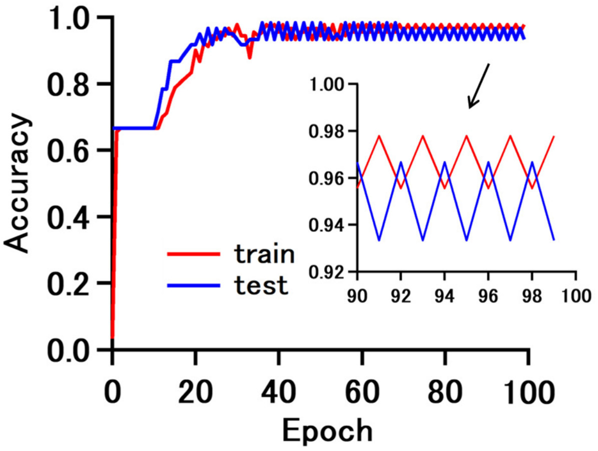

Supplementary Fig. 12: A PyTorch-based class used to construct a 4×5×3 ANN model, and its the training curve of accuracy in classifying Iris dataset using the same RMSprop optimizer with a learning rate of 0.005.

4.4 Experimental robustness analysis for Iris classification

After training, we obtained the voltage weight at each heater (corresponding to Fig. 5 in the main text and see Supplementary Fig. 11). The learned voltage weights can be loaded into the device anytime for repeating verification. Then, we can intentionally introduce a random bias error to the weight and measure the variation in the accuracy and MSE. The error was introduced as $\mathbf{V} = \mathbf{V}(1 + \mathbf{r}\Delta)$ ($\mathbf{V}$ is the voltage weight vector, $\mathbf{r}$ is a vector of random number in [-1,1] and Δ is the bias error). For Iris classification, both the train and test sets were examined using the voltage weights obtained by both BFO and RMSprop. The changes in classification results induced by this random deviation are shown in

Supplementary Fig. 13. When the bias error <3%, there is no obvious degradation in accuracy for both BFO and RMSprop. For the bias error >3% and <7%, BFO seems having more robust training results than RMSprop since it has a slower decrease in accuracy and a slow increase in MSE. Further increase in the bias error >7% induces large random variations in accuracy and quick increase in MSE. Therefore, <3% bias control precision is required to avoid accuracy degradation. Supplementary Fig. 14 shows the confusion matrix of Iris classification at 3% and 7% bias errors on the BFO curves in Supplementary Fig. 13(a). At the 3% bias error, the labels 1 and 2 are wrongly recognized. But when increasing the error to 7%, the label 0 (=Setosa) seems suffering larger influences than the labels 1 (=Versicolor) and 2 (= Virginica).

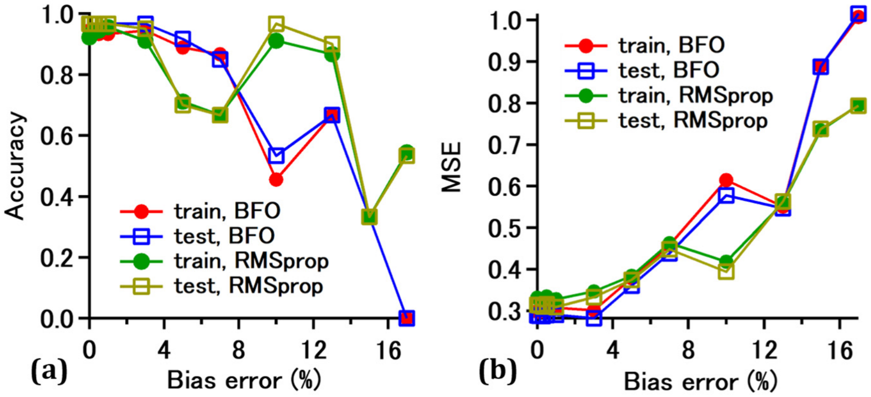

Supplementary Fig. 13: Experimental results of Iris classification robustness to the bias error. Measured accuracy (a) and MSE (b) with increasing the bias error for both train set (90 samples) and test set (60 samples). The voltage weights obtained by both BFO and RMSprop were examined.

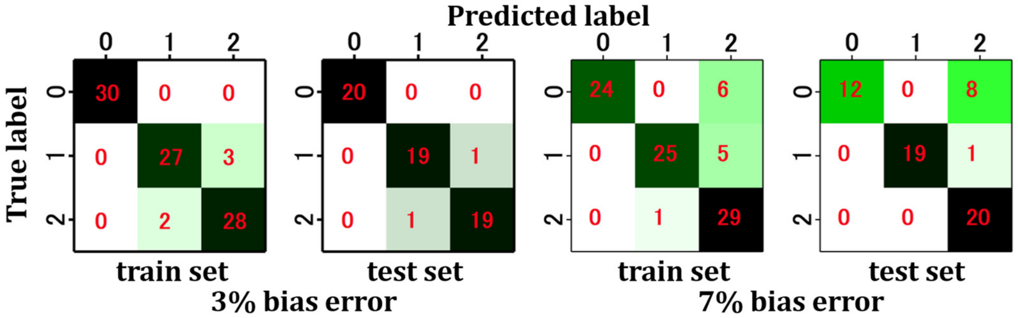

Supplementary Fig. 14: Confusion matrix of Iris classification using the BFO-trained voltages with bias errors.

4.5 On-chip training experiment with drop out for Iris classification

We did all the same things (on-chip training and robustness analysis) as did above for Iris classification with drop out (only using ports 1, 3, 5 for the output vector and discarding other ports, as explained in the main text and above in Supplementary Section 3.4). The experimental training curves are shown in Supplementary Fig. 15(a). After training, the accuracies were verified to be ~98.9% and ~98.3% for the train and test sets, respectively, as seen from the confusion matrices in Supplementary Fig. 15(b). In average, the accuracy is 98.7% for all 150 samples, and this experimental value is comparable to that (97.3%) (of complex photonic neural networks) in Ref. [8]. Next, we performed robustness analysis similarly as did in Supplementary Fig. 13(a). Supplementary Fig. 15(c) shows the bias error induced accuracy degradation. Compared to that without drop out, this accuracy is more sensitive to the bias

error, >1% deviation causing a rapid decrease in accuracy. Therefore, with drop out, more precise voltage control is required. The experimental accuracies are consistent with the simulated ones in Supplementary Fig. 15(d).

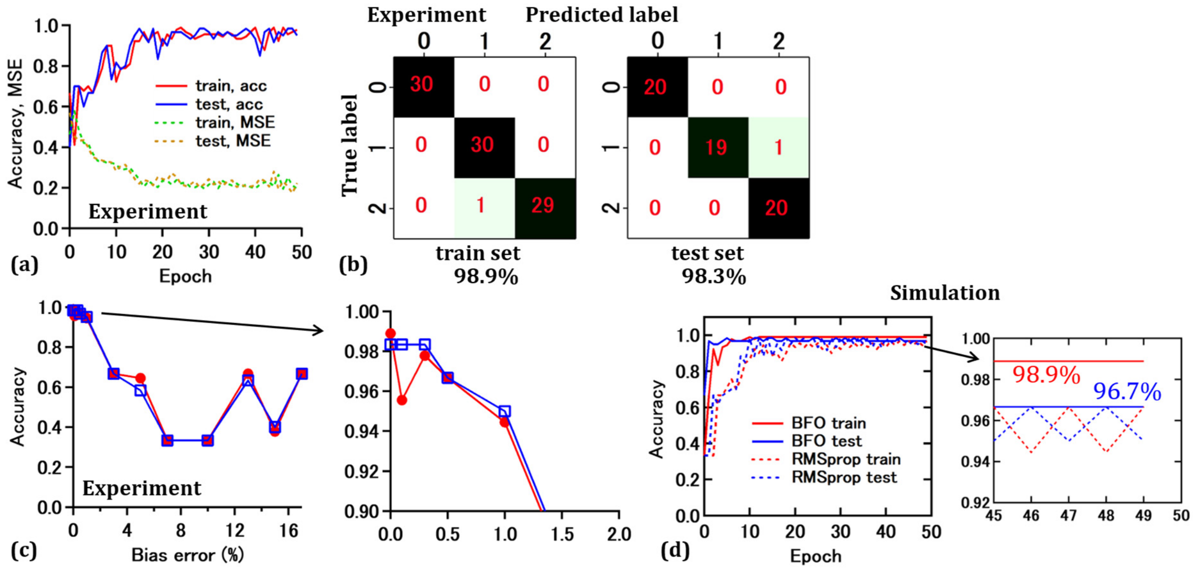

Supplementary Fig. 15: Iris classification experiment with drop out by BFO training. (a) Accuracy and MSE vs epoch. (b) Confusion matrix for the train (90 samples) and test (60 samples) sets after training. (c) Accuracy robustness to the bias error. (d) Simulated accuracy for the case of drop out (from Supplementary Fig. 24(c)).

5. Reproducibility and long-time stability

Reproducibility includes two aspects: sample reproducibility and measurement reproducibility. We selected another chip on the same wafer and completed wire bonding packaging. Using this wire bonding chip, we repeated all experiment as done in the previous experiment using 40-pin probes and obtained reproduceable experimental results in this revised manuscript, as shown below.

5.1 Long-time stability of Iris classification results

After BFO training, the learned voltage weights were re-loaded into the device repeatedly within several days and performed verification for the samples in both train and test sets. As shown in Supplementary Fig. 16, the accuracy and MSE are almost same as those of just after training, indicating the long-time stability of classification results.

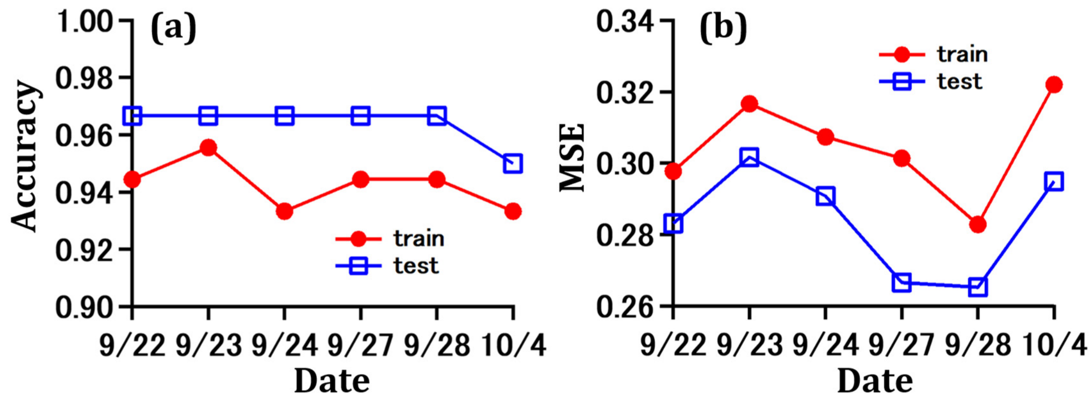

Supplementary Fig. 16: Long-time stability of Iris classification performance. The accuracy (a) and MSE (b) were checked by repeatedly re-loading the voltage weights (in Supplementary Fig. 11) learned by BFO.

5.2 Long-time stability of port reconfiguration experiment

The MSE in Supplementary Section 5.1 reflects the long-time stability of the optical power distribution among all ports for the Iris classification. Here, we show the stability of each individual port after the light is reconfigured to each single port by BFO. For example, as shown in Supplementary Fig. 17 (a), we can automatically reconfigure the device to maximize the optical power at the ports 5 and 7. The start and end curves are before and after training. We did this for all ports and measured the MSE, as shown in Supplementary Fig. 17(b). For each port, we re-loaded the obtained voltage weights into the device and checked the MSE variations. We did not observe any degradation in MSE within one-week verification for each port, indicating the control reproducibility and device stability, as shown in the right figure of Supplementary Fig. 17(b).

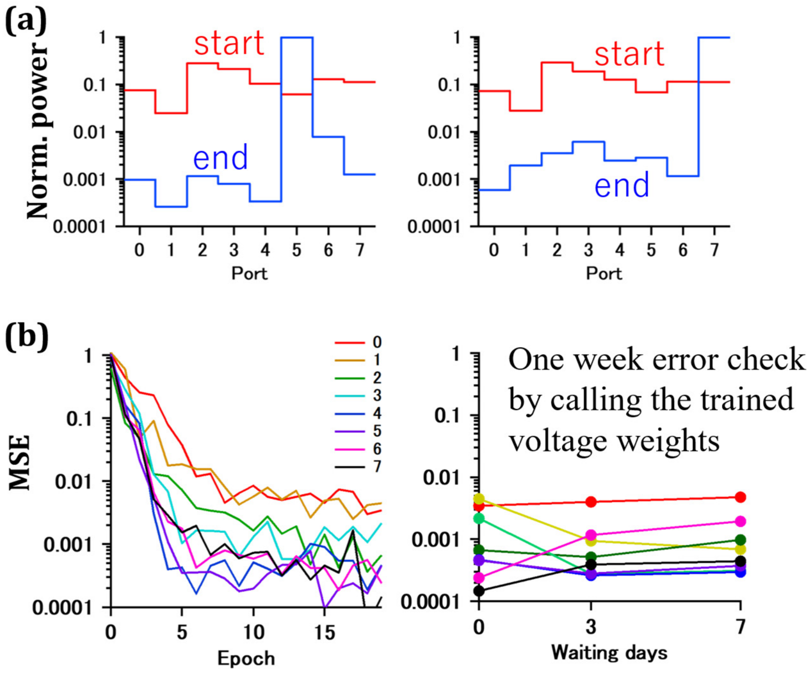

Supplementary Fig. 17: (a) Automatic reconfiguration to route the light to the port 5 and 7 by BFO. The start curve is before training and the end curve is after training of 20 epochs. (b) Residual MSE for each port and its repeated measurement results within one week.

5.3 Measurement environment and device structure

We performed all experiments at room-temperature lab environment, without any thermal management steps, as seen in Supplementary Fig. 18. In previous experiment, we used 40-pin probes to contact the pads, however, in current experiment, we used a wire bonding chip instead. The wire bonding chip offers much better stability. As seen from the cross-section figure in Supplementary Fig. 18, the thermal equilibrium is mainly established at local surface since the cap layer is thin. Thus, inter-heater thermal interference is small, which is same as our previous large-scale optical switches in Ref. [9]. More important, because we implement direct on-chip training by BFO, this algorithm can automatically take all factors into training process, therefore, the final solution after training already includes any possible influences from uncertainties of heaters. This also explains the long-time reproduceable experimental results given in Supplementary Sections 5.1 and 5.2.

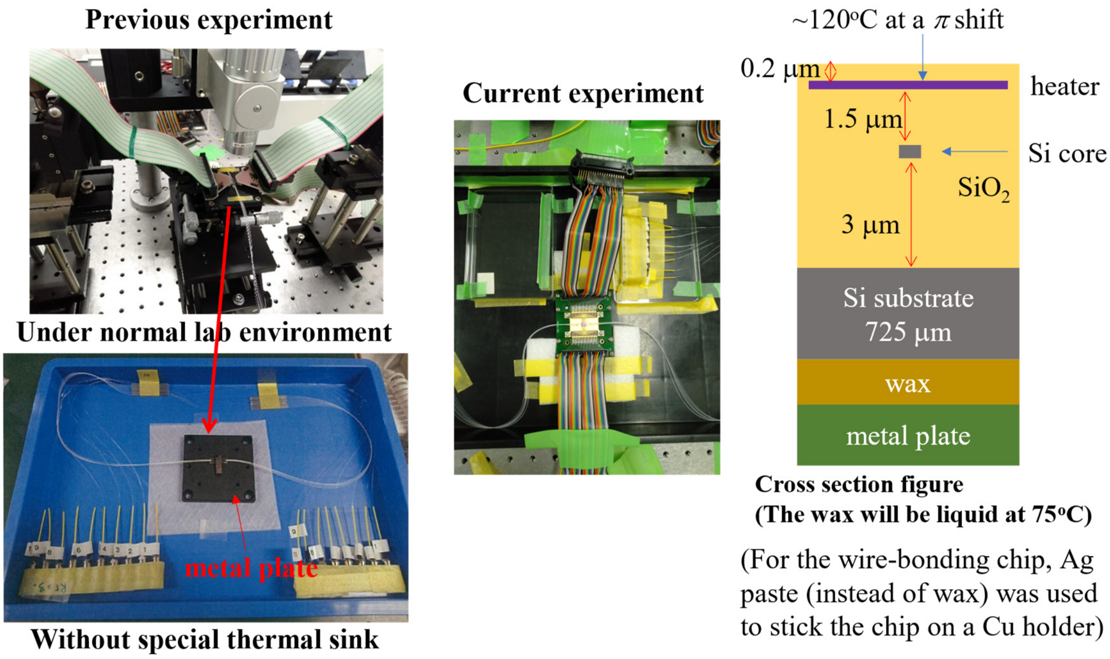

Supplementary Fig. 18: Measurement environment and device figures of previous experiment and current experiment using a wide bonding chip. Cross section of the device.

5.4 Robustness to device imperfection

We examine the influence of device imperfection (e.g., fabrication error) on reproducibility by simulation. In silicon photonics, one of the most sensitive components is the directional coupler (DC) (one MZI has two DCs). For switching application, it is required to be as close as to 3 dB ideally to guarantee low crosstalk as seen in Refs. [9, 10]. For classification application, to clarify the influence of DC errors, we introduce a maximum deviation δ(10%) as seen in the equation in Supplementary Fig. 19 which means the deviation ratio from $\pi/4$. This deviation is randomly generated for all DCs. Taking XOR as the example, as shown in Supplementary Fig. 19, even with an error, the final MSE in training is almost same as that without error. Increasing the error does not monotonously increase the MSE and the training is still successful even with errors. As seen from the optical propagation inside the device, the final XOR ports (the port 1 denotes 0, the port 5 denotes 1) can also be correctly trained out, showing almost the same separation result. This is different from switching application, because for switching, the light always goes along a single path that is greatly influenced by the DC error in each MZI along the path; but for classification, the light serves for a multiple path interference. Even with the errors inside the device, the training will re-figure out a different multiple path interference that can convey the same classification information at the output. This can be seen from the light propagation in Supplementary Figs. 19(a) and 19(b), in which the inside interference paths are different for with and without errors, however, the final classification results are same. For Iris classification, the final MSE even becomes smaller if including random errors, as seen in Supplementary Fig. 19(c). Therefore, the classification application is more robust to fabrication errors and the reproducibility of classification devices is in principle higher than other silicon photonic devices consisting of DCs such as switches and micro-rings. BFO was used to obtain the results in Supplementary Fig. 19.

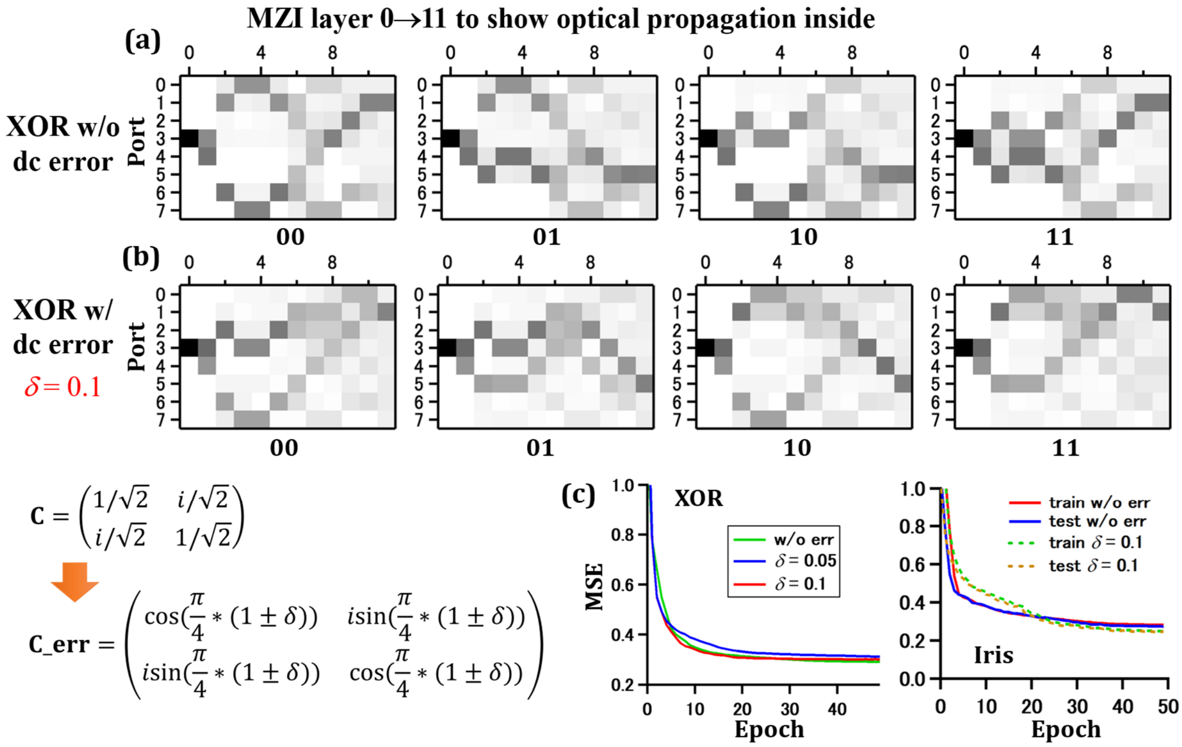

Supplementary Fig. 19: The optical power mapping shows the simulated optical propagation inside the device for XOR separation (a) without the DC error and (b) with the DC error $\delta = 0.1$, δ is the directional coupler (DC) deviation ratio defined in the equation. (c) Comparison on MSE with and without including δ for XOR and Iris.

6. Scalability examination by MNIST simulation

Here, we investigate the scalability of accuracy and power of the projection-based method by simulating MNIST (handwritten digit dataset [11]) classification. This simulation is classifying the *k*-space patterns of MNIST images obtained by FFT preprocessing [6, 7]. We prepared four architectures G1–G4 in Supplementary Fig. 20 for this case. Since the data extracted from *k*-space patterns are complex values, they are input via 1×1 MZI with an inner phase shifter ϕ and an external phase shifter θ, which is related to the *k*-space data x as $\phi = \pm\arcsin(|x|)$ and $\theta = \pm(\arcsin(-\mathrm{real}(x)/|x|) - \phi)$ (minus sign when $\mathrm{imag}(x) < 0$). Thus, the architecture G1 is linear in the complex space, which can be treated as a reference. We use architecture G2–G4 to form nonlinear mapping in the complex space by cascading two times data input with an intermediate VMM. For understanding these schemes, we take a two-element vector $\mathbf{x} = (x_1, x_2)$ as an example to explain the mapping functions. Here we omit coefficients for simpleness and then the projected vector is $\mathbf{x}' = (x_1(x_1+x_2), x_2(x_1+x_2))$. For dot product with another vector $\mathbf{v}' = (v_1(v_1+v_2), v_2(v_1+v_2))$, the result is $\langle \mathbf{x}', \mathbf{v}' \rangle = x_1v_1(x_1+x_2)(v_1+v_2) + x_2v_2(x_1+x_2)(v_1+v_2) = (x_1v_1+x_2v_2)(x_1+x_2)(v_1+v_2) = \mathbf{x}\cdot\mathbf{v}(x_1v_1+x_2v_2+x_1v_2+x_2v_1) = \mathbf{x}\cdot\mathbf{v}(\mathbf{x}\cdot\mathbf{v} + \mathbf{x}\cdot\mathbf{U}\cdot\mathbf{v})$ where $\mathbf{U} = \begin{pmatrix} 0 & 1 \\ 1 & 0 \end{pmatrix}$. Thus, these mapping functions are quadratic-like ones in the complex space. One-hot optical power vector $\mathbf{y_{10}}$ is extracted from the output ports 0→9 and the argmax($\mathbf{y_{10}}$) is used to mark the correct label. The training uses the normalized $\mathbf{y} = \mathbf{y_{10}}/\|\mathbf{y_{10}}\|$ to calculate the MSE loss, which offers a faster convergence than other normalization methods and loss functions [7]. We used 500 images for each digit (loading images from the MNIST data file *train-*

images.idx3-ubyte(http://yann.lecun.com/exdb/mnist/) in the default order and saved images according to their labels. For each digit, we used the first 500 images); thus, there are 5000 images in total, 3000 for training, 2000 for testing. All samples are trained with a batch size of 200 and 100 epochs. For each architecture in Supplementary Fig. 20, the total parameters (weights) are indicated by summing the phase shifters in the order of Splitter + PS + VMM + PS + VMM. The scalability is examined by increasing the input parameters and scale, within an achievable range for current silicon photonic platforms. The maximum-scale VMM is consisted of 16×(32+31) = 1008 MZIs, which is achievable as seen in [9, 12]. RMSprop was adopted here for training, for comparison with ANN.

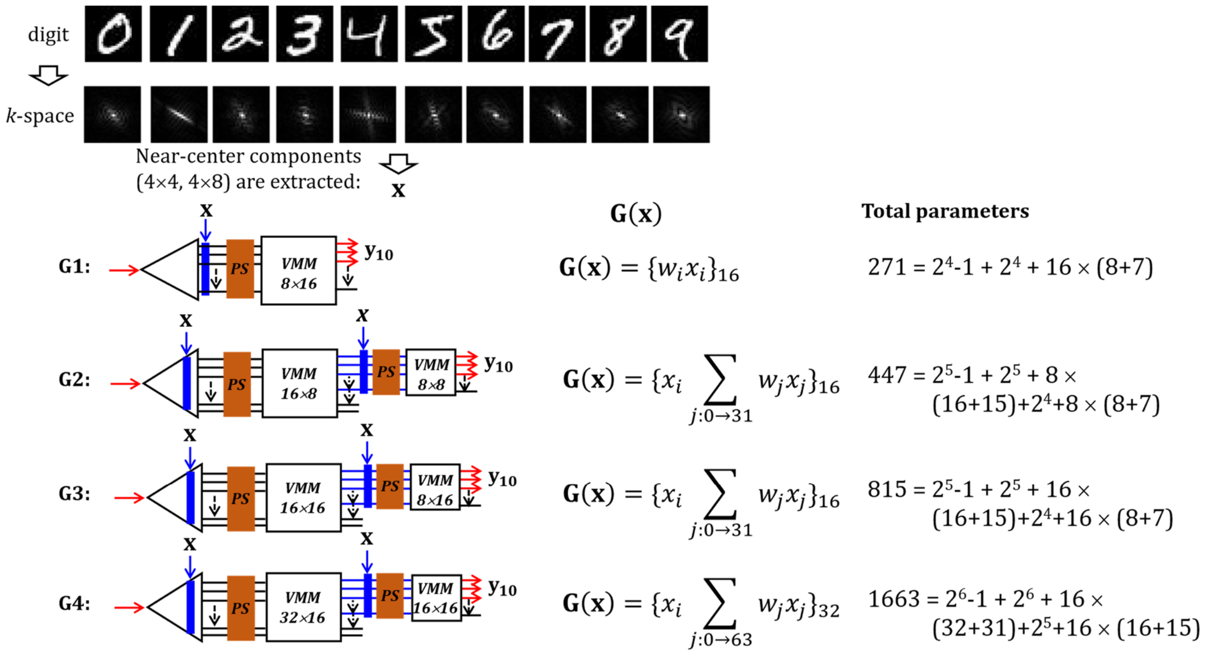

*VMM is denoted by the MZI mesh of the maximum row number × the half column number, e.g., $N\times M$ means MZI numbers = $N\times M$ (outer set) + $(N-1)\times M$(inner set)

Supplementary Fig. 20: Four architectures to generate mapping functions for classifying the FFT-processed *k*-space patterns of MNIST. G1-G3 take 16 components for input and G4 takes 32 components for input.

6.1 Scalability of accuracy and power

Supplementary Fig. 21(a) shows the accuracies of four architectures in Supplementary Fig. 20. For G1, this is a linear mapping since **x** is in complex domain, thus, its accuracy (~86%) is close to that (~85%) of the linear photonic ANN [6]. From G1 to G3, the accuracy can be enhanced to 90–91% due to the projection effect. This is a benchmark to obtain >90% accuracy with only <900 parameters and without traditional nonlinear activation functions used in ANN. For G4, ~96.6% training accuracy and ~94% testing accuracy can be achieved with 1663 parameters. For ANN [6, 8], it is known that such an accuracy cannot be achieved without nonlinear activation functions, evidencing a different principle from ANN. For 16 and 32 input components, ~5% and ~4% accuracy enhancement can be achieved, respectively, by implementing nonlinear projection in G2–G4 compared to the linear one in G1 (as seen by comparing Supplementary Fig. 21 (a) and Fig. 22). For quantitative description, the scalability of accuracy has a dependance of log(N^0.13) for training and log(N^0.09) for testing to the total parameter number N.

With increasing the scale, the increase in power is related to the increase of total phase (summing up all phase shifters). As shown in Supplementary Fig. 21(b), the total phase shift is linearly increasing with N. The total power depends on what kind of the phase shifter is used. If using the same thermo-optic one (assuming P_π = 15 mW), ~5 W power is required in G4, which is still much lower than current GPU or CPU. If using other phase shifters with high energy efficiency such as pin-type [13], PCM-type [14–16], or MEMS-type [12], the total power can be further decreased. For example, the pin-type phase shifter using carrier injection usually has a π-shift power P_π ~2–3 mW, only 1/5 of the thermo-optic one; then the total power is expected about 1 W. The PCM and MEMS types can offer powerless standby states. For G4 in Supplementary Fig. 21(a), after training, the confusion matrices were verified, indicating the accuracies of 97.9% and 94.0% for the train and test sets, respectively, as shown in Supplementary Figs. 21(c) and 21(d). (This accuracy 97.9% of the train set is slightly higher than that (~96.6%) in Supplementary Fig. 21(a) because that Supplementary Fig. 21(a) was checked before each epoch of updating weights. Thus, after the final accuracy was checked, there was one more time weight updating, for which the accuracy is that in Supplementary Fig. 21(c)). Thus, by using only passive circuits, this principle can achieve accuracies comparable/slightly higher to/than those up to ~93% for 2 layers in [6], 90.5 in [8], ~93.4% in [17], ~90% in [18], and lower than 98% in [7] that adopted 64 input components and parallel nullification calibration method.

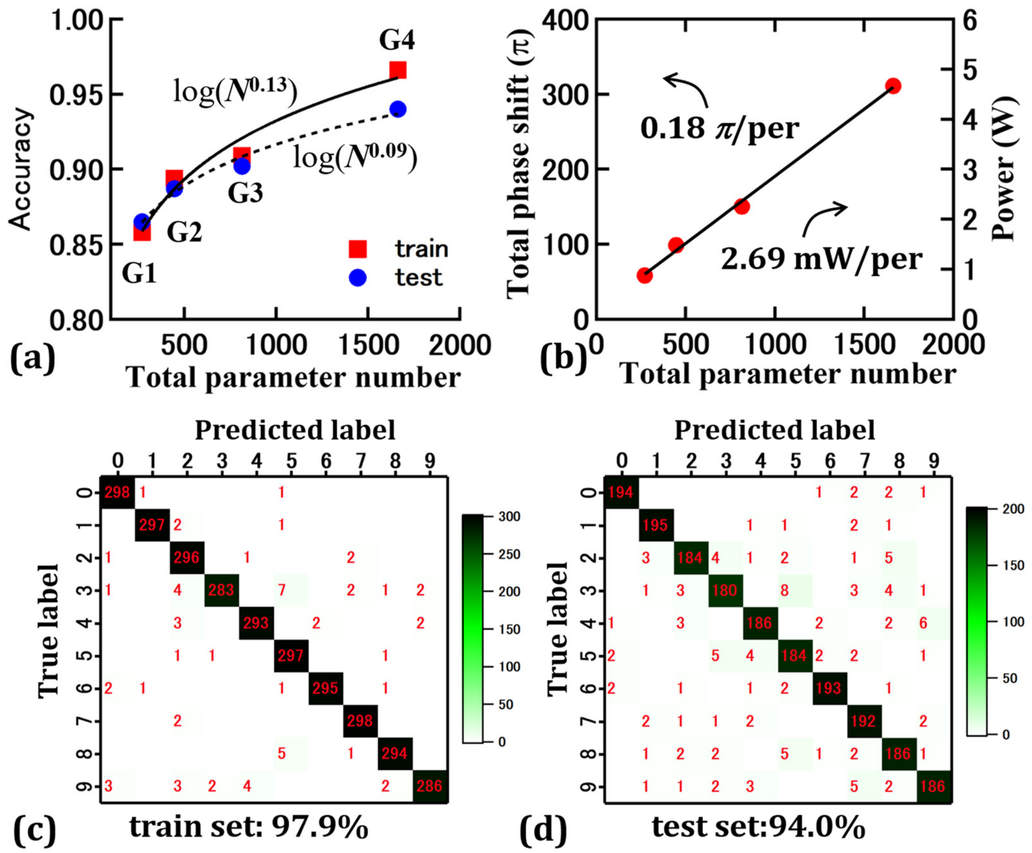

Supplementary Fig. 21: Scalability examination by MNIST simulation. (a) Scalability of accuracy. (b) Scalability of the power (absolute sum of total phases) assuming using thermo-optic phase shifter with a π-shift power P_π= 15mW. Confusion matrices verified for (c) the train set and (d) the test set after training for G4 in (a).

6.2 Influence of MZI layer number of MZI-mesh-based VMM

The VMM adopts the Clements' topology [5] as seen in Fig. 1a in the main text. Further adding more duplicate MZI layers (column) will overfit the matrix transformation and obviously will not degrade the performance. The lowest requirement of MZI layers is investigated for MNIST classification using the structure G1. As shown in Supplementary Fig. 22, the accuracy first increases with increasing the layer number and subsequently becomes almost constant after reaching its maximum, which means that further increasing the layer number will not contribute to accuracy enhancement anymore once the layer number is sufficient. By comparing the results of G3 and G4 in Supplementary Fig. 21(a) with the saturated maximum accuracies in Supplementary Figs. 22(a) and 22(b), respectively, we can see that nonlinear projection can contribute to ~4–5% accuracy enhancement.

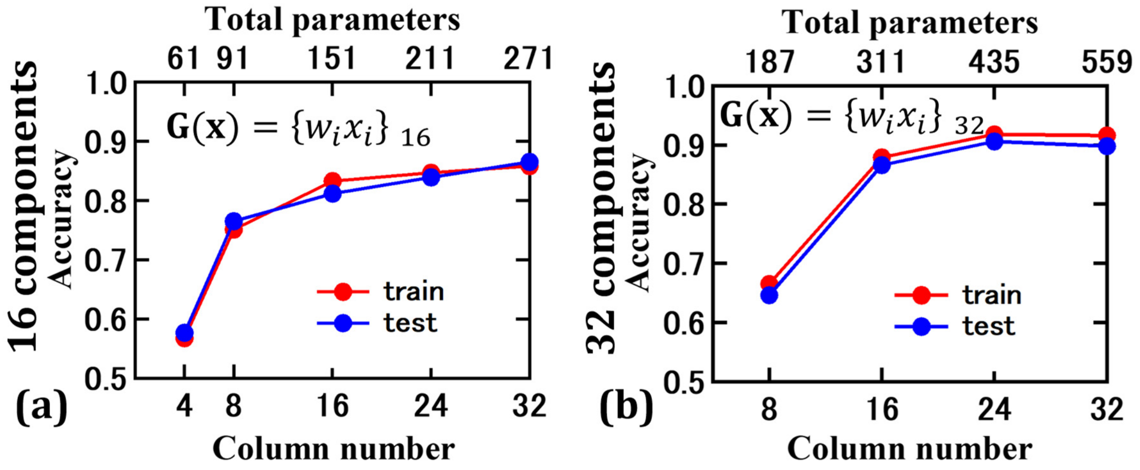

Supplementary Fig. 22: Accuracy in relation to the MZI layer number (column number) of VMM for MNIST classification using: (a) 16-components input and (b) 32-components input. The column number here is counted as indicated in Fig. 1a in the main text, including two MZI sets (the column number = 2M, M is used in Supplementary Fig. 20). Total parameters of the entire structure are also indicated in the top axis.

6.3 With or without external phase shifters for MZI

Further adding more parameters such as increasing the layer number (see Supplementary Section 6.2) or adding external phase shifters (see schematic in Supplementary Fig. 23) for each MZI obviously will not degrade the classification performance, but the total number of training parameters will be increased, hence increasing the training time. In all above simulations no external phase shifters were used and the obtained training results are the specific solutions at θ = 0. Here we compare the classification results with and without external phase shifters. After adding external phase shifters to all MZIs, we repeat the simulation in Supplementary Fig. 22(a). As seen in Supplementary Fig. 23, with external phase shifters, we can notice that (1) the accuracy can be enhanced for insufficient column numbers (e.g., 4, 8); (2) the accuracy reaches its maximum with fewer column number; (3) the maximum (saturated) accuracy has no increase compared to that without external phase shifters in Supplementary Fig. 22(a). The observed slight decrease in accuracy after 24 columns is not true decrease since the increase in total parameter number needs more epochs to achieve better convergence, while here all simulations were fixed to 100 epochs.

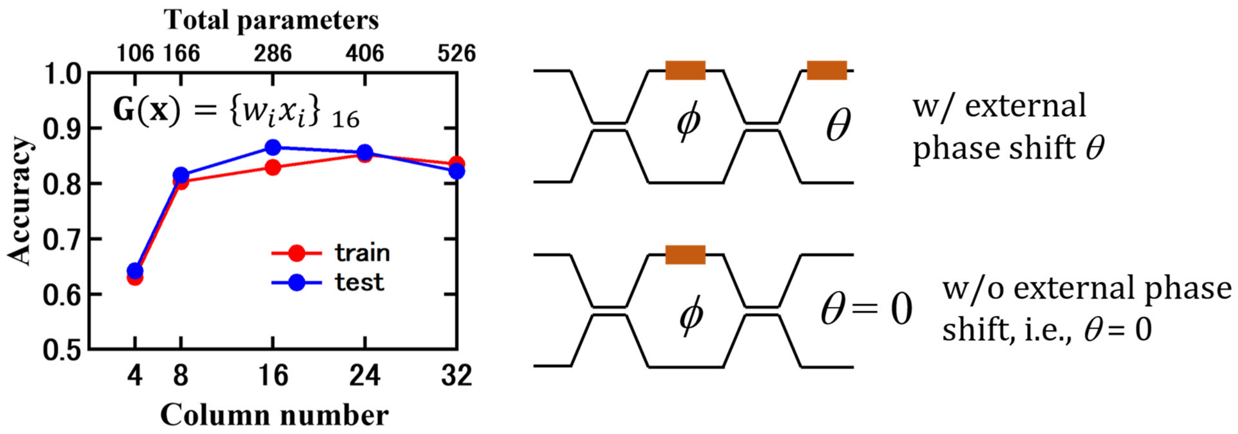

Supplementary Fig. 23: Accuracy in relation to MZI layer number (column number) of VMM with external phase shifters for all constituted MZIs. This simulation is same as did in Supplementary Fig. 22(a) except for adding θ as additional training parameters. Total parameters are indicated in the top axis. At the right the schematic depicts the MZI with and without the external phase shifter θ.

7. BFO vs RMSprop for training four tasks

Experimental comparison between these two algorithms (BFO and forward propagation using RMSprop optimizers) has been discussed in the main text and Supplementary Sections 3 and 4 above. Here we compare them by simulation for all three tasks: XOR, Iris, Iris with drop out, and MNIST. The simulation was done in a similar way as experiment. The device was treated as a black box for which the algorithm did not know any intermediate parameters inside, like running the algorithm on a real chip. The algorithm used the same code as used in experiment. The only difference is that in simulation the phase is the training parameter, but in experiment, the voltage is the training parameter. Thus, the algorithm in simulation can be implemented in an exact same way in experiment without requiring doing additional works such as preparing optical error vector or chip calibration [8]. The PPC device here for XOR and Iris is same as that used in simulating the nonlinear dataset classification in Fig. 6 in the main text. All training curves including the accuracy and MSE are summarized in Supplementary Fig. 24. For XOR in Supplementary Fig. 24(a) and Iris in Supplementary Fig. 24(b), BFO and RMSprop give almost same accuracy and MSE after 50 epochs. For Iris in Supplementary Fig. 24(c) with drop out (see Supplementary Section 4.5), BFO offers a higher accuracy (as seen the enlarged figure in Supplementary Fig. 15(d)) and a smaller MSE than RMSprop. For MNIST in Supplementary Fig. 24(d), BFO and RMSprop have almost same accuracy and MSE after 100 epochs. But RMSprop converges faster than BFO. This is because for all situations in Supplementary Fig. 24, BFO only uses 10 bacteria which is not enough for a large parameter space. Despite of only 10 bacteria, using a smaller step (BFO (2) in Supplementary Fig. 24(d)) can result in a faster convergence, much close to the curves of RMSprop. Thus, more bacteria and smaller steps are preferred for training in a large parameter space for BFO.

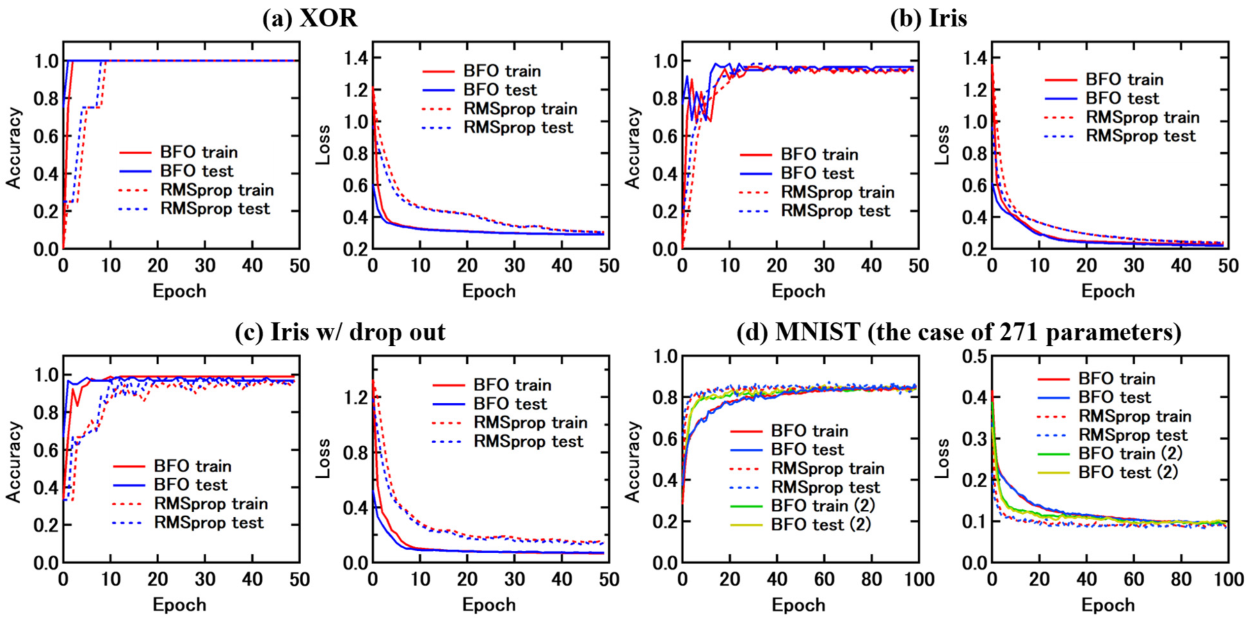

Supplementary Fig. 24: Comparison between BFO and RMSprop (forward propagation) algorithms for training three benchmarks. Training curves of accuracy and MSE for (a) XOR, (b) Iris, (c) Iris with drop out, (d) MNIST using the structure G1 in Supplementary Fig. 20. BFO and RMSprop adopted the same conditions as mentioned in the main text (see Methods) except for (d) where two cases of BFO adopt smaller adaptive steps of $\Delta\phi = 0.01\delta$ and 0.005δ (BFO (2)) for comparison. δ is the MSE loss.

8. Benchmark comparison to a previous paper

We summarize all benchmarks in this work together with those demonstrated in a complex photonic neural network in Ref. [8] (Zhang et al. *Nat. Commun.* **12**, 457 (2021)) in Supplementary Fig. 25. Note that in this work, we implement not only photonic inference, but also on-chip training, instead of using a computer neural network model and calibration. For these traditional benchmarks, our work offers comparable performances to this paper and demonstrated functions such as combinational logic operations (XOR-AND (half adder), OR-NAND) in experiment.

	On-Chip training?	XOR	Other logic (AND, OR, NAND)	Combinational logic	Iris	MNIST	Nonlinear dataset (Acc.)		
							circle 2-class	moon 2-class	spiral 3-class
Zhang et al Nat. Comm. 12, 457 (2021)	No (the decomposition and implementation of the weight matrices on chip)	Ok (exp)	Ok (exp)	-	0.974(exp)	200 samples train 93.1% 200 samples test 90.5% (inference exp)	1	-	-
Our work	Yes (both training & inference are done on chip)	Ok (exp)	Ok (exp)	XOR-AND, OR-NAND (exp)	0.983(exp) 0.967(exp)	3000 samples train 96% 2000 samples test 94% (on-chip training sim)	1(sim)	1(sim)	0.9 (sim)

Supplementary Fig. 25: Comparison between this work and a previous paper for various benchmarks. For Iris, in our work, the experimental testing accuracies 98.3% and 96.7% are obtained with and without drop out.

9. BFO source code and videos of BFO algorithm

Notes: (1) BFO engine used in our experiment and simulation is shown in Supplementary Fig. 26. Codes are made based on Python using PyTorch (//pytorch.org/) and other open packages.

(2) Three Supplementary Videos of BFO algorithm are uploaded for understanding this algorithm.

BFO source code

```
def BFOEng(self, data, yt, loss, delta, B1, Jlast, step, iserr, op, opn):
    for nc in range(5): #10
        for sb in range(B1.size(0)):
            dv = torch.rand(B1.size(1))*2.0 - 1.0
            Bc = B1[sb,:].detach().clone()
            Bc = Bc + step * dv/torch.square(dv).sum().sqrt()
            CopyB2M(self, Bc)
            yo, loss, acc, y, op, opn = self(data, yt, iserr, op, opn)
            for m in range(20):
                if (loss < Jlast[sb]):
                    Jlast[sb] = loss.detach().clone()
                    Bc = Bc + step * dv/torch.square(dv).sum().sqrt()
                    CopyB2M(self, Bc)
                    yo, loss, acc, y , op, opn= self(data, yt, iserr, op, opn)
                else:
                    break
            Jlast[sb] = loss.detach().clone()
            B1[sb,:] = Bc.detach().clone()
        sort = torch.argsort(Jlast)
        B2 = B1.detach().clone()
        B1[0:int(B1.size(0)/2),:] = B2[sort[0:int(B1.size(0)/2)],:]
        B1[int(B1.size(0)/2):int(B1.size(0)),:] = B2[sort[0:int(B1.size(0)/2)],:]
        Jtemp = Jlast.detach().clone()
        Jlast[0: int(Jlast.size(0)/2)] = Jtemp[sort[0:int(Jlast.size(0)/2)]]
        Jlast[int(Jlast.size(0)/2):int(Jlast.size(0))] = Jtemp[sort[0:int(Jlast.size(0)/2)]]
```

Supplementary Fig. 26: Source code of BFO algorithm engine used in this work.

References for Supplementary file

[1] Ren, J. ANN vs SVM: which one performs better in classification of MCCs in mammogram imaging. *Knowledge-Based Systems*, **26**, 144–153 (2012).

[2] Smola, A. J. and Scholkopf, B. A tutorial on support vector regression. *Statistics and Computing*, **14**, 199–222 (2004). (2004 Kluwer Academic Publishers).

[3] Schölkopf, B. & Smola, A. J. *Learning with Kernels: Support Vector Machines, Regularization, Optimization, and Beyond* Ch. 1&2 (MIT Press, London, 2002).

[4] Steinwart, I. and Christmann, *A. Support vector machines: Information Science and Statistics Series* (Springer, 2008).

[5] Clements, W. R. et al. Optimal design for universal multiport interferometers. *Optica* **3**, 1460–1465 (2016).

[6] Williamson, I. A. D. et al. Reprogrammable Electro-Optic Nonlinear Activation Functions for Optical Neural Networks. *IEEE J. Sel. Top. Quant. Elec.* **26**, 7700412 (2020).

[7] Pai, S. et al. Parallel programming of an arbitrary feedforward photonic network. *IEEE J. Sel. Top. Quant. Elec.* **26**, 6100813 (2020).

[8] Zhang, H. et al. An optical neural chip for implementing complex-valued neural network. *Nature Communications*. **12**, 457 (2021)

[9] Tanizawa, K. et al. Ultra-compact 32×32 strictly-non-blocking Si-wire optical switch with fan-out LGA interposer. *Opt. Express* **23**, 17599–17606 (2015).

[10] Suzuki, K. et al. Strictly Non-Blocking 8 × 8 Silicon Photonics Switch Operating in the O-Band. *J. Lightwave*

Technol. **39**, 1096–1101 (2021).

[11] LeCun, Y., Bottou, L., Bengio, Y. & Haffner, P. Gradient-based learning applied to document recognition. *Proc. IEEE* **86**, 2278–2324 (1998).

[12] Seok, T. J. et al. Wafer-scale silicon photonic switches beyond die size limit. *Optica* **6**, 490–494 (2019).

[13] Dupuis, N. et al. Nanosecond photonic switch architectures demonstrated in an all-digital monolithic platform. *Opt. Lett.* **44**, 3610–3612 (2019)

[14] Feldmann, J. et al. Parallel convolutional processing using an integrated photonic tensor core. *Nature* **589**, 52–58 (2021).

[15] Wu, C. et al. Programmable phase-change metasurfaces on waveguides for multimode photonic convolutional neural network. *Nature Communications* **12**, 96 (2021).

[16] Shastri, B. J. et al. Photonics for artificial intelligence and neuromorphic computing. *Nature Photonics* **15**, 102–114 (2021).

[17] Lin, X. et al. All-optical machine learning using diffractive deep neural networks. *Science* **361**, 1004–1008 (2018).

[18] Xu, X. Y. et al. 11 TOPS photonic convolutional accelerator for optical neural networks. *Nature* **589**, 44–51 (2021).
