# Supplementary material for: On-chip bacterial foraging training in silicon photonic circuits for projection-enabled nonlinear classification
Source: Nat Commun. 2022 Jun 30;13:3261. doi: 10.1038/s41467-022-30906-3 (PMC9247170; doi:10.1038/s41467-022-30906-3)
Supplement: Supplementary file 3 — Description of Additional Supplementary Files [file 41467_2022_30906_MOESM3_ESM.docx]

**Description of Additional Supplementary Files**

File name: Supplementary Video 1

Description: A video to show using the BFO algorithm to find the minimum of a 10-dimension sphere function (video displays in 2D parameter space).

File name: Supplementary Video 2

Description: An experimental video of BFO training for the XOR separation experiment.

File name: Supplementary Video 3

Description: An experimental video of BFO training for automatic port configuration as shown in Supplementary Fig. 17.
